# Healthcare-associated respiratory syncytial virus infections in children’s hospitals

**DOI:** 10.1017/ash.2022.174

**Published:** 2022-05-16

**Authors:** Lisa Saiman, Susan Coffin, Larry Kociolek, Danielle Zerr, Aaron Milstone, Margaret Aldrich, Celibell Vargas, Morgan Zalot, Megan Reyna, Amanda Adler, Annie Voskertchian, Emily Egbert, Luis Alba, Madelyn Ruggieri, Yoonyoung Choi

## Abstract

**Background:** Little is known about the impact of healthcare-associated respiratory syncytial virus (HA-RSV) in hospitalized children. To address this gap, we assessed the epidemiology and clinical impact associated with HA-RSV in a multiseason, multicenter study. **Methods:** During respiratory viral seasons 2016–2017, 2017–2018, and 2018–2019, we retrospectively identified HA-RSV cases in hospitalized children 72 hours after admission or within 48 hours of discharge in readmitted patients. Due to reduced availability of testing for non–SARS-CoV-2 viruses during the first year of the COVID-19 pandemic, the 2019–2020 season was excluded. We initiated prospective HA-RSV surveillance during the 2020–2021 season and continued surveillance through November 2021 due to the unusual interseasonal RSV community outbreak. We determined demographic and clinical characteristics of HA-RSV cases and explored possible outcomes associated with RSV including transfer to the pediatric ICU and escalation of respiratory support from day −2 to day +4 (day 0 was the day of RSV detection). We explored the timeframe of day −2 to day +4 because events during this timeframe could be attributed to RSV infection. Respiratory support escalation was defined as change from room air to supplemental oxygen, increase in fraction of inspired oxzygen (FiO_2_) on same respiratory support modality, or change from noninvasive to invasive support. **Results:** Were identified 86 HA-RSV cases: 20 (23.3%) from 2016–2017, 26 (30.2%) from 2017–2018, 34 (39.5%) from 2018–2019, and 6 (7%) from October 2020–November 2021. HA-RSV was diagnosed a median of 14 days (IQR, 8–45) after admission. Moreover, 29 (33.7%), 31 (36.0%), and 26 (30.2%) cases were aged 60 months during these, respective seasons. Also, 33 (38.4%) had >3 comorbid conditions, most commonly gastrointestinal (n = 33, 38.4%), respiratory (n = 28, 32.6%), and/or congenital–genetic disorders (n = 28, 32.6%). However, 9 (10.5%) had no comorbid conditions. From day −2 to day +4, 15 children (17.4%) were transferred to the PICU and 38 (49.3%) of 77 evaluable cases required respiratory support escalation, most commonly supplemental oxygen delivered by nasal cannula (n = 15, 19.5%). Furthermore, 11 patients (14.3%) required invasive support. **Conclusions:** HA-RSV was associated with use of healthcare resources, including the need for respiratory support escalation and/or transfer to intensive care. From October 2020 to November 2021, lower numbers of HA-RSV were observed. The reasons for this are unknown, but potentially occurred in parallel to markedly reduced RSV in the community and may have resulted from visitor restrictions, which included no siblings and/or universal masking by hospital staff and visitors.

**Funding:** Funding for this research was provided by Merck Sharp & Dohme, a subsidiary of Merck & Co.

**Disclosures:** None

